# Intratumoral heterogeneity in microsatellite instability status at single-cell resolution

**DOI:** 10.1016/j.isci.2026.114860

**Published:** 2026-02-05

**Authors:** Harrison Anthony, Cathal Seoighe

**Affiliations:** 1 School of Mathematical and Statistical Sciences, University of Galway, Galway, Ireland; 2 The Research Ireland Centre for Research Training in Genomics Data Science, Galway, Ireland

**Keywords:** Cancer, Cell biology, Genetics

## Abstract

Intratumoral heterogeneity complicates the interpretation of single-test biomarkers. Microsatellite instability (MSI) is one such biomarker, which is used to guide immune checkpoint inhibitor treatment by classifying samples as having high microsatellite instability (MSI-H) or as microsatellite stable (MSS). However, it is unknown whether MSI itself is a heterogeneous phenomenon. To test this, we curated data from several single-cell RNA sequencing studies with clinical MSI status and developed a computational pipeline that quantifies intratumoral heterogeneity in MSI. Out of 49 individuals, 15 showed evidence of divergence in MSI status between clusters of cancer cells, and most had distinct MSI-H and MSS subclones. These results question the use of MSI as a binary biomarker, and we hypothesize that accounting for heterogeneity could improve its use as a predictive biomarker. Further studies are required to determine the frequency of MSI heterogeneity at the population level and whether it can have clinical implications.

## Highlights

• Novel computational pipeline quantifies ITH and MSI at single-cell resolution
• 15 out of 49 individuals have evidence of ITH in MSI
• Both MSI-H and MSS individuals have tumors with distinct MSI-H and MSS subclones
• Findings challenge the current binary classification of MSI status

## Graphical abstract

**Figure:**
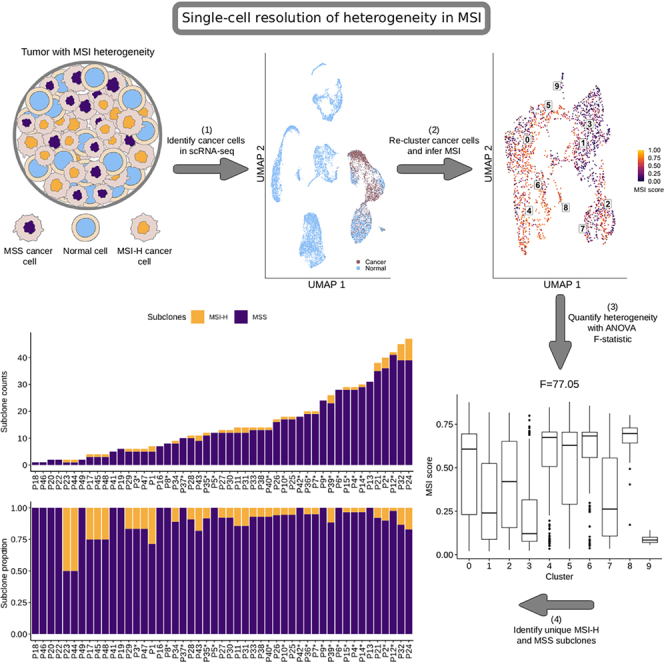

## Introduction

Subclonal diversity within a tumor is a critical consideration in cancer research and treatment. The overall diversity found in a single neoplasm is called intratumoral heterogeneity (ITH). While ITH was first conceptualized to be genetic in nature,^1^ it is now used to describe genetic, epigenetic, and phenotypic differences between subclones.^2^ The diversity within a tumor is important because ITH has been linked to poor patient outcomes, therapy resistance, and relapse.^3^^,^^4^ Furthermore, biomarkers that rely on single-sample tests can be susceptible to sampling bias when ITH is present.^5^^,^^6^ While its origins are still debated,^7^ one well-known driver of ITH is genome instability.^8^

Genome instability is a hallmark of cancer, characterized by a higher rate of accumulation of mutations during replication, typically due to deficiencies in DNA repair genes.^9^ The two most common forms of genomic instability are at the chromosomal level, where instability is characterized by aneuploidy and chromosomal aberrations,^10^ and at the microsatellite level, where short tandem repeats expand and contract in a mutator phenotype manner.^11^ The latter, referred to as microsatellite instability (MSI), is hypothesized to be the result of a deficient mismatch repair (dMMR) pathway and is commonly used as a biomarker to help guide immune checkpoint inhibitor (ICI) treatment. This is done by classifying cancers as either having high microsatellite instability (MSI-H) or as being microsatellite stable (MSS).^12^ The classification is normally carried out using a single-sample test that compares five microsatellite markers between a tumor and paired-normal sample.^13^^,^^14^ While the interplay between chromosomal instability and ITH is well defined and explored,^15^^,^^16^^,^^17^ the relationship between MSI and ITH is less clear.

Up to this point, most research on MSI and ITH has been framed around how MSI can impact and shape the variation present within a tumor. Most studies in this area have focused on specific mutations^18^^,^^19^ and the immune cell types present in the tumor microenvironment (TME),^20^^,^^21^ with the latter being crucial to precision medicine efforts. Researchers have shown that MSI-H cancers have a “hot” microenvironment with an abundance of tumor-infiltrating lymphocytes,^22^ and that they respond well to anti-PD-1 therapy, which prevents T cell exhaustion.^23^ Despite the successes of using MSI status to guide anti-PD-1 therapy, there are still challenges to its adoption as a predictive biomarker.

Some issues remain with the use of MSI status as a predictive biomarker as researchers have reported cases of low treatment response rates and intrinsic treatment resistance when using MSI status to guide anti-PD-1 therapy.^22^^,^^24^^,^^25^ One possible explanation for this is ITH as it has been linked to therapy resistance^3^^,^^4^ and is known to complicate the interpretation of clinical biomarkers.^5^^,^^6^ While researchers have studied cell types present in the TME, little is known about whether MSI itself is a heterogeneous phenomenon. Although some case studies exist that documented cases of ITH in MSI status,^26^^,^^27^^,^^28^^,^^29^^,^^30^^,^^31^ the question of whether MSI itself is frequently a heterogeneous phenomenon, with some subclones displaying MSI while others do not, has yet to be examined in detail. This warrants further investigation as it may ultimately lead to improved biomarker performance.

The current literature suggests that subclonality of MSI status is relatively rare or entirely absent,^32^^,^^33^ but that is not always the case. There are many examples of individuals not only with discordant MSI statuses between the primary tumor site and metastases^26^^,^^27^^,^^28^^,^^29^ but also between multiple sites in the primary tumor.^30^^,^^31^ While these are small case studies, they provide anecdotal evidence for cancers comprising MSI-H and MSS subclones. However, there has, as yet, been no attempt to evaluate the frequency with which this occurs. Furthermore, a detailed examination of heterogeneity requires an assessment of MSI at the single-cell level with next-generation sequencing (NGS), not with the traditional methods of PCR and IHC used in these case studies, as these are limited to detecting clear spatial heterogeneity.

Here, we aimed to address these gaps through an analysis of published single-cell datasets that include paired clinical MSI status. To do this, we developed a custom Snakemake^34^ pipeline that identifies MSI-H cells and uses novel methods to assess levels of heterogeneity and have made this pipeline available as an open-source, scalable resource to the scientific community. We evaluated the pipeline by mixing varying numbers of MSI-H and MSS cells from different samples. Applying this framework, we show evidence of heterogeneity in MSI status at the single-cell level and estimate its prevalence in the curated data. We also examine the nature of MSI heterogeneity through a detailed investigation of single-cell data from two individuals – one classified as MSI-H and the other MSS through PCR/IHC tests.

## Results

### Computational pipeline distinguishes high microsatellite instability and microsatellite stable individuals and captures intratumoral heterogeneity

**Figure 1:**
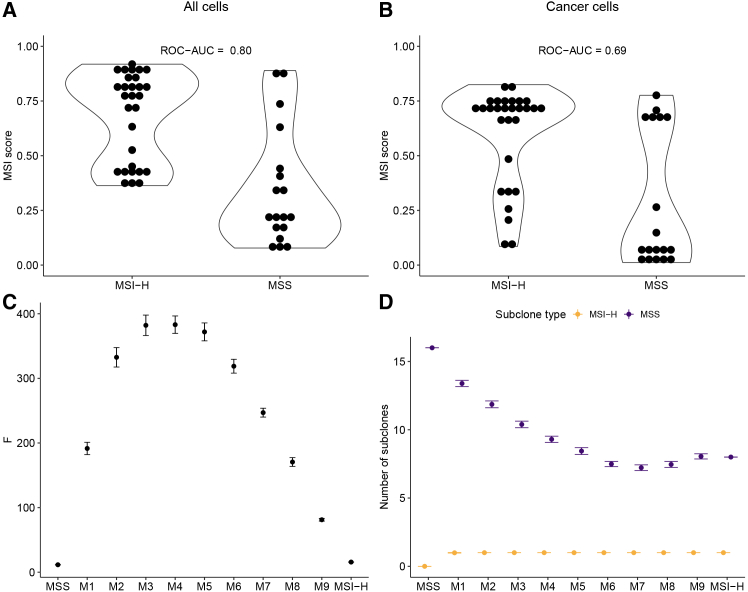
MSIsensor-RNA and simulation results (A and B) Boxplots show the distribution of (A) MSI score for individuals calculated using the aggregate expression of all cells, and (B) MSI score for individuals calculated using the aggregate expression of only cancer cells. (C and D) Also shown are the mean values of (C) the F-statistic and (D) the number of subclones for the different cell mixes shown on the x axes (with increasing proportions of MSI-H cells ranging from 0.1 in mix M1 to 0.9 in mix M9). The error bars in (C) and (D) correspond to plus/minus twice the standard error around the mean. The MSS and MSI-H samples in panels C and D are the obtained values for all cells in those samples and do not represent an average. See Figure S1 showing ROC and precision-recall curves for MSIsensor-RNA as well as Tables S1 and S2 which contain summary statistics and the raw results for the mixing experiments, respectively.

To determine whether MSIsensor-RNA could distinguish between MSI-H and MSS individuals, we ran it on the aggregate expression of all cells and again on only the cancer cells for each individual. As expected, MSI-H individuals generally had higher MSI scores than MSS individuals, and MSIsensor-RNA was able to broadly distinguish between the two groups (Figures 1A and 1B; Figures S1A and S1B). These results were seen for both aggregated expression of all cells and only cancer cells, but subsetting down to only cancer cells yielded lower MSI scores. There were also disagreements between PCR/IHC MSI status and MSIsensor-RNA score with several MSS individuals having relatively high MSI scores and several MSI-H individuals having low MSI scores (Figures 1A and 1B).

Next, we simulated different levels of heterogeneity to determine how well our pipeline captured ITH in MSI status. For this purpose, we simulated different levels of heterogeneity, ranging progressively from pure MSS cells to pure MSI-H cells by mixing together samples from two individuals comprised of homogeneously MSI-H cancer cells and homogeneously MSS cancer cells (Tables S1 and S2). As expected, the more homogeneous samples (MSS, mix M1, mix M9, and MSI-H in Figure 1C) had low F-statistic values, while mixtures with more equal proportions of MSI-H and MSS cells (mixes M3-M5 in Figure 1C) had high F-statistic values. Increasing the proportion of MSI-H cells until mix M7 resulted in an overall reduction in the number of MSS subclones identified (Figure 1D; Tables S1 and S2), and there was one MSI-H subclone that was consistently detected after the proportion of MSI-H cells was 0.1 (mixes M1-M9). Together, these results show that the F-statistic is sensitive to ITH and that the number of subclones can be consistently identified across replicates, providing useful context to the heterogeneity.

### High microsatellite instability and microsatellite stable individuals have evidence of intratumoral heterogeneity in microsatellite instability status

**Figure 2:**
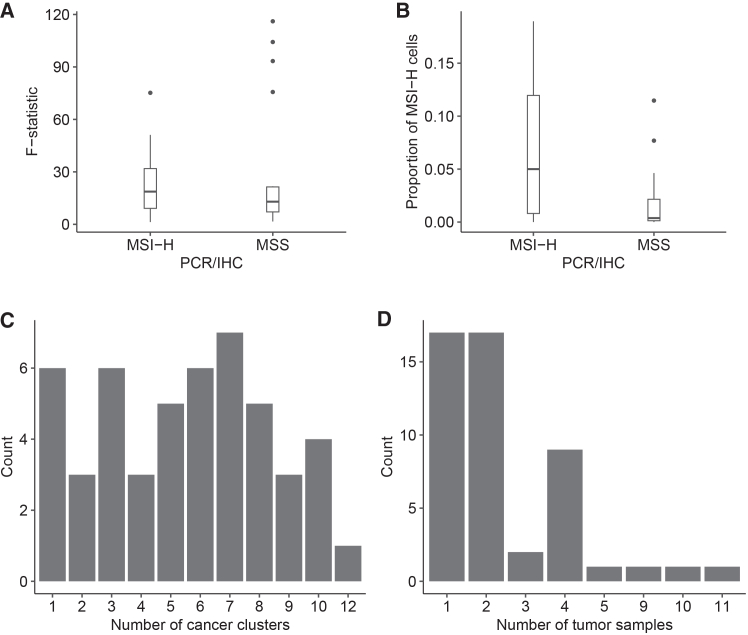
Distributions of summary statistics in single-cell RNA sequencing data (A and B) Boxplots show the distribution of (A) F-statistics grouped by PCR/IHC MSI status and (B) the proportion of MSI-H to MSS cells grouped by PCR/IHC MSI status. (C and D) Histograms display the frequency of (C) the number of cancer cell clusters and (D) the number of tumor samples for all individuals. See Table S3, which includes the ANOVA test results for each individual.

In order to assess heterogeneity in MSI status, we first calculated F-statistics (see STAR Methods) based on clusters of cancer cells and identified subclones based on CNV patterns. We found that MSI-H and MSS individuals both had evidence of heterogeneity in MSI. In total, 15 of 49 individuals showed evidence of divergence in MSI status between distinct clusters of cancer cells (F > 25; Table 1 and Figure 2A). Several individuals had very large estimates of heterogeneity based on F-statistics (75.20–116.10), with most of these individuals being originally deemed to be MSS, and one originally deemed to be MSI-H from a PCR or IHC test. In contrast, the lowest F-statistics (1.30–1.68) were found in MSI-H and MSS individuals, and the ANOVA tests were not statistically significant in either case (*p* > 0.05; Table S3). This was also seen in most other individuals with fewer than three cancer cell clusters (Table 1; Table S3).

**Table 1:** Individual summary statistics and subclone information

Individual	Clusters	F	Samples	MSI-H cells	MSS cells	MSS	MSI-H	PCR/IHC
CRC2783	4	17.15	3	40	211	5	2	MSI-H
CRC2786	10	75.67	4	101	2182	36	4	MSS
CRC2787	2	1.62	2	4	121	5	1	MSS
CRC2794	6	10.23	4	3	939	28	1	MSS
CRC2795	6	8.17	4	0	367	7	0	MSS
CRC2801	7	4.8	5	1	1019	7	0	MSS
CRC2803	8	16.04	4	5	619	19	1	MSS
CRC2810	3	1.68	4	1	155	7	0	MSS
CRC2811	8	19.63	4	1	1255	7	0	MSS
CRC2816	7	8.21	4	2	585	17	1	MSS
CRC2817	7	10.62	10	56	442	12	2	MSI-H
CRC2821	11	100.85	9	240	9698	41	1	MSS
CRC2829	9	10.04	2	0	1147	7	0	Unknown
CRC2841	8	93.35	11	5	1392	29	1	MSS
CRC2899	9	20.28	4	3	1444	28	1	MSS
P11	3	6.94	1	0	119	7	0	MSI-H
P12	3	3.02	1	11	92	3	1	MSI-H
P14	1	NA	1	1	20	7	0	MSI-H
P15	3	2.25	1	0	139	7	0	MSI-H
P17	1	NA	1	0	36	7	0	MSI-H
P18	10	30.89	1	58	1633	35	3	MSI-H
P19	2	1.3	1	0	41	7	0	MSI-H
P21	1	NA	1	3	27	1	1	MSI-H
P23	10	29.07	1	200	1785	39	8	MSI-H
P24	7	75.2	1	22	732	17	1	MSI-H
P25	7	34.94	2	13	630	16	1	MSI-H
P26	6	5.09	1	12	427	12	1	MSI-H
P27	5	11.26	2	4	385	10	1	MSI-H
P28	5	51.15	1	4	192	5	1	MSI-H
P29	5	10.99	1	3	380	12	1	MSI-H
P30	6	26.69	1	62	434	12	2	MSI-H
P31	10	18.71	2	149	1833	39	6	MSI-H
P32	7	19.41	2	3	370	13	1	MSI-H
P33	6	15.71	1	24	301	8	1	MSI-H
SC024	5	21.77	2	4	338	11	1	MSS
SC027	8	20.28	2	3	769	19	1	MSS
SC029	4	5.78	2	0	300	7	0	MSS
SC035	6	47.22	2	37	415	13	1	MSI-H
SC040	9	116.1	4	82	1067	23	3	MSS
SC041	5	10.01	2	32	279	13	1	MSS
SC042	3	31.24	2	0	141	7	0	Unknown
SC043	7	6.75	2	0	813	7	0	MSS
SC044	8	31.9	3	58	412	9	2	MSI-H
SRR23490337	1	NA	1	3	31	1	1	MSI-H
SRR23490338	2	7.66	1	11	70	3	1	MSI-H
SRR23490339	1	NA	1	0	18	7	0	MSI-H
SRR23490340	4	27.42	1	24	190	5	1	MSI-H
SRR23490341	3	48.02	1	14	74	3	1	MSI-H
SRR23490342	1	NA	1	0	48	7	0	MSI-H

This table contains the summary statistics for each individual included in the analysis. The clusters column refers to the number of unique cancer clusters, and F is the ANOVA F-statistic used to measure heterogeneity in those clusters. The samples column describes the number of samples each individual had for the analysis, and the MSI-H and MSS cells column describes the number of cell types for that individual. The MSS and MSI-H columns refer to the number of microsatellite stable and microsatellite instability high subclones for each individual. The original IHC/PCR status for each individual is included and was established in previous studies (Table 2). Any NA values represent F-statistics that could not be calculated due to fewer than 2 clusters of cancer cells being present.

**Figure 3:**
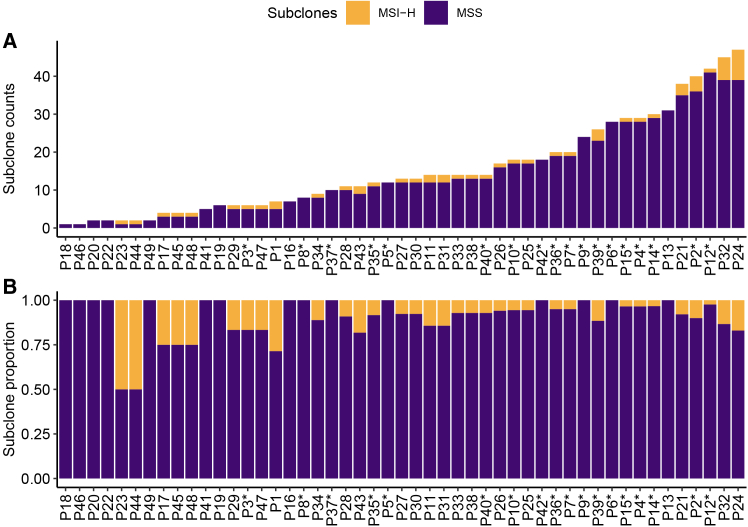
MSI-H and MSS subclone compositions (A and B) Stacked bar plots of (A) the number of subclones for each individual in the analysis and (B) the proportion of subclone types for each individual. Individuals who had a PCHR/IHC test result of MSS are indicated with an asterisk.

In general, MSI-H and MSS individuals had similar distributions of summary statistic values but with several outliers having large F-statistic values among the MSS individuals (Figure 2A) and MSI-H individuals having higher proportions of MSI-H cells (Figure 2B). Interestingly, nearly every individual in the analysis had both MSI-H and MSS subclones, and a larger proportion of MSS subclones (Figures 3A and 3B; Table 1). The exceptions were two individuals who each had a subclone proportion of 0.5 (Figure 3B), but they had very few cancer cells and too few cancer clusters to calculate an F-statistic for comparison (Table S3). Those with the most MSI-H subclones, six and eight, were originally determined to be MSI-H, but one MSS individual also had four MSI-H subclones. Independent of MSI status, the distribution in number of clusters across individuals was relatively even, and most individuals had two or fewer samples used in the clustering process (Figures 2C and 2D).

### Single-cell level resolution of heterogeneity in one high microsatellite instability and one microsatellite stable individual

**Figure 4:**
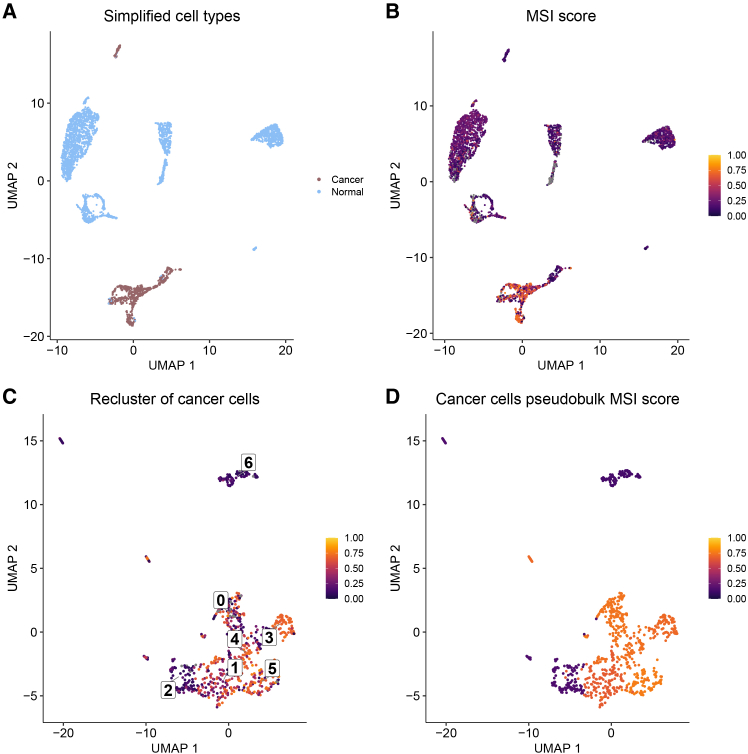
Clustering of cells for the MSI-H individual (A–D) UMAP plots for MSI-H individual P24 show (A) tumor versus normal cell classification, (B) MSI scores for each cell, (C) MSI scores for re-clustered cancer cells, and (D) MSI score for the aggregated pseudobulk expression of each cancer cell cluster. Any gray colors indicate an NA value. See also Tables S4, S6, and S8, which contain the results of Tukey HSD analysis between all cancer cell clusters, differential gene expression analysis between all cancer cell clusters, and differential gene expression analysis between MSI-H and MSS cells, respectively.

**Figure 5:**
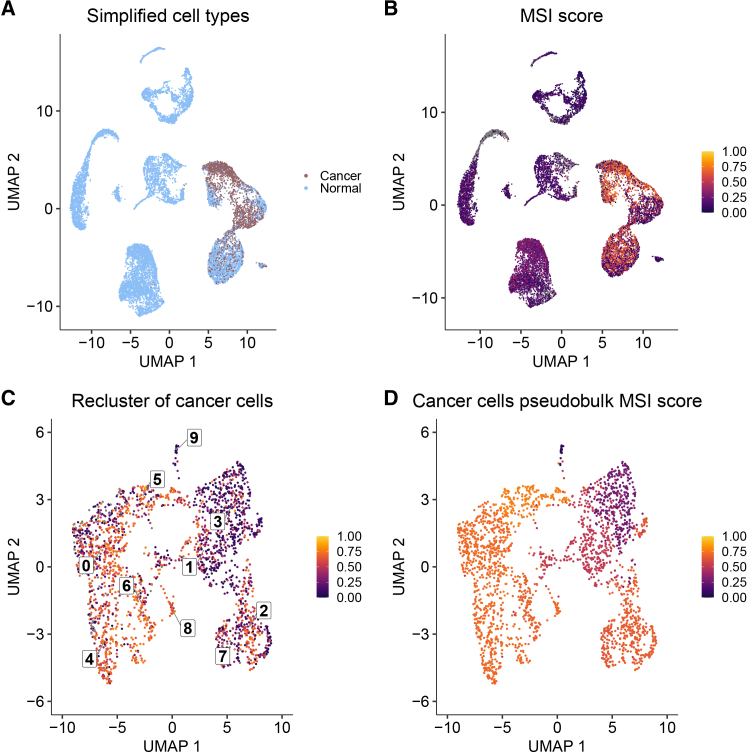
Clustering of cells for the MSS individual (A–D) UMAP plots for MSS individual CRC2786 show (A) tumor versus normal cell classification, (B) MSI scores for each cell, (C) MSI scores for re-clustered cancer cells, and (D) MSI score for the aggregated pseudobulk expression of each cancer cell cluster. Any gray colors indicate an NA value. See also Tables S5, S7, and S9, which contain the results of Tukey’s HSD analysis between all cancer cell clusters, differential gene expression analysis between all cancer cell clusters, and differential gene expression analysis between MSI-H and MSS cells, respectively.

We selected two individuals (P24 and CRC2786) with relatively high F-statistics and many MSI-H subclones to illustrate the heterogeneity in MSI that is evident from single-cell RNA-Seq data (Figures 4 and 5). The MSI-H individual, P24, had good overlap in cells classified as cancer with scATOMIC (Figure 4A) and those with high MSI scores determined by MSIsensor-RNA (Figure 4B). The re-clustered cancer cells appear to cluster by MSI score; notably, clusters two and three (Figure 4C). Those larger differences in the clusters were also seen in the pseudobulk analysis of the re-clustered cancer cells (Figure 4D).

The MSS individual, CRC2786, also had good overlap between the cells determined to be cancerous and those with a high MSI score; however, there was less separation of cancer and normal cells in this individual (Figures 5A and 5B). Similarly, in the re-clustered cancer cells, cells with higher and lower MSI scores were somewhat more intermingled than for the MSI-H individual, although clusters three and four seem to be predominantly MSS and MSI-H, respectively (Figure 5C). This result is recapitulated in the pseudobulk analysis, with cluster three showing a low MSI score and cluster four a much higher one (Figure 5D).

### Significant differences in microsatellite instability score and gene expression between clusters of cancer cells

**Figure 6:**
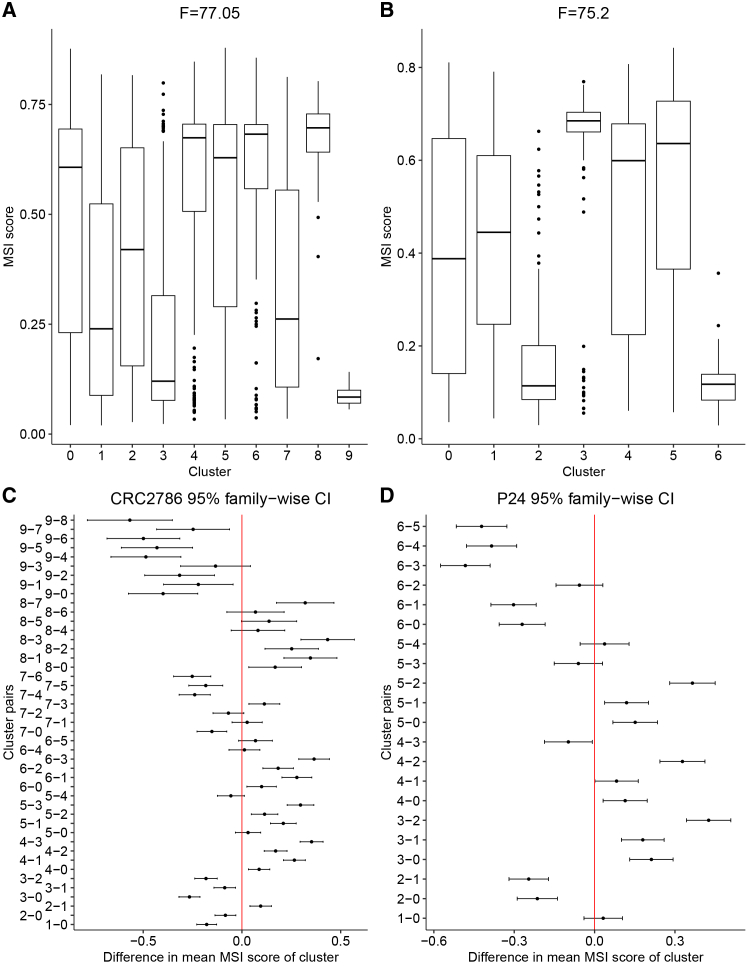
Distribution of MSI scores and difference in means for cancer cell clusters (A–D) Boxplots show the distribution of MSI scores for each cluster of cancer cells in (A) individual CRC2786 and (B) individual P24. Also shown are the 95% confidence intervals for the difference in mean MSI scores between each cluster pair for (C) individual CRC2786 and (D) individual P24.

We examined MSI ITH in CRC2786 and P24 further by assessing differences between clusters of cancer cells. We found both individuals to have many clusters with significantly different MSI scores (Figure 6), and several genes showed differences in expression between clusters and between cells identified as MSI-H and MSS (Figures S2 and S3). Both individuals had clusters with high and low MSI scores (Figures 6A and 6B). These differences were found to be statistically significant (*p* < 0.05 using a Tukey HSD test; Tables S4 and S5). We found that 35 cluster pairs for CRC2786 had significantly different MSI scores and 17 cluster pairs for P24 (Figures 6C and 6D) were significantly different.

Within the clusters of cancer cells, gene expression was also significantly different between clusters (Figures S2A and S3A; Tables S6 and S7), and between the MSI-H and MSS cells in those clusters (Figures S2B and S3B; Tables S8 and S9). The top five differentially expressed genes for each cluster of cancer cells for each individual were retained for analysis, as well as the top 50 differentially expressed genes between MSI-H and MSS cells. Individual CRC2786 had three genes: *MALAT1, EEF1A1,* and *SH3BGRL3* in common between those differentially expressed between clusters and between cells with different MSI status; Figures S2A and S2B). P24, on the other hand, had one gene, *PCLAF,* that was differentially expressed between clusters and between MSI-H and MSS cells. When comparing the differential expression analyses for both individuals, we found three genes: *BMX*, *LRMP,* and *SH2D6* that were differentially expressed between clusters, and two genes (*TYMS*, *OXCT1*) in common that differentiated MSI-H and MSS cells.

## Discussion

In our study, we showed that MSI status can be heterogeneous at the single-cell level and provided a pipeline to measure that heterogeneity with the clustering of cancer cells and CNV-based subclone analysis. These results contrast with the assumption that is commonly made, both in research and in clinical practice, that MSI is dichotomous. While this assumption has proven useful, enabling MSI-H to be used as a biomarker for ICI treatment,^35^ overall responder rate has been reported to be as low as 31%.^22^ We hypothesize that this could be explained, at least in part, by the heterogeneity in MSI-H individuals, which a binary classification fails to take into account.

Single-sample tests (like the ones used to assign MSI status) are susceptible to under-sampling bias when spatial ITH is present.^3^ This would require multi-sample, multi-regional tests to improve classification, as MSI-H and MSS cells could be concentrated in different regions of the tumor. We could not resolve whether the heterogeneity we identified was organized spatially because we did not have access to spatial data. However, there is some evidence that this could be the case. One previous study found that MSI-H colorectal cancers had spatial ITH of specific frameshift mutations in several mononucleotide repeats, but this regional ITH was determined to be the result of disease progression and not related to an MSI-H phenotype.^36^ Similarly, another study also found discrepancies between IHC and PCR tests when sampling different regions of MSI-H cancers, suggesting that a multi-sample biopsy would be more appropriate when testing for MSI.^26^

Both studies, however, used PCR or IHC tests and likely missed the level of heterogeneity we discovered by analyzing single-cell data. A future study with spatially resolved single-cell transcriptomics^37^ would provide an opportunity to determine if ITH in MSI is structured regionally and whether this has any clinical relevance. For example, if we found that MSI-H and MSS cells were regionally clustered and separated from one another, this would suggest that the single-region, single-sample biopsy technique currently in use to assess MSI clinically is inadequate.

One of our primary findings, that both MSI-H and MSS individuals had a mixture of MSI-H and MSS cells, is supported by the findings of another study.^38^ Similarly to our study, they also found that MSI-H and MSS individuals had evidence of ITH in MSI using single-cell sequencing data; however, their methodology, which involved clustering cells based on the gene-set enrichment of MSI-H and MSS signatures, did not identify any MSS individuals with only MSS cells. Our pipeline was able to find examples of MSS individuals comprising MSS cells only, which would make sense given that MSI is a relatively rare trait, and it would be unlikely to be present in every MSS individual in a study cohort. This is due to the main difference between our methods, as we test individual cells for microsatellite instability, whereas Zhao et al.^38^ labeled cells as MSI-H at the pseudo-bulk level with gene-set enrichment guided cell clustering. Our study is also unique as we aimed to quantify ITH and provide our pipeline in an open-access format.

Our finding that nearly every MSI-H and MSS individual had MSI-H and MSS subclones has not yet been reported in other studies; however, two case studies that infer subclonality of dMMR status from discordant IHC test results have been reported.^27^^,^^39^ Even though dMMR and MSI-H technically refer to different phenomena, MSI is considered to be the byproduct of dMMR, and both are predictive of ICI treatment efficacy. Combined with our findings, these case studies provide insights that could help explain reports of 30% or more of MSI-H cancers having primary resistance to single-agent ICI’s.^24^^,^^40^

A treatment regimen for an MSI-H cancer would potentially miss one or more MSS subclones, leaving behind a population of cells that would not respond in the same way to immunotherapy. This is because a tumor with coexisting MSI-H and MSS subclones would have a different type of TME shaped by immune cells and the PD-1/PD-L1 pathway. The key difference is that a tumor consisting mostly of MSI-H subclones will have a higher neoantigen load due to an abundance of frameshift mutations,^41^^,^^42^ and consequently, a “hotter” TME characterized by many tumor-infiltrating lymphocytes.^22^ In turn, this leads to an upregulation of PD-L1 in tumor and stromal cells in the TME, causing T cell dysfunction,^23^ and allows cancer cells to escape immunosurveillance.^43^ Investigation of the impact of MSI heterogeneity on treatment and the TME would benefit from longitudinal data consisting of tumor samples before and at several time points after treatment from individuals with and without heterogeneity in MSI. This type of study would be warranted by our results, as we provide a plausible mechanism for treatment resistance, which is not currently given adequate consideration.^40^

Our computational pipeline is the first to identify and quantify heterogeneity in MSI status at the single-cell level. We built the pipeline around MSIsensor-RNA and scATOMIC, two pan-cancer, machine learning-based approaches. The combination of these programs may give rise to some potential issues. Naturally, as both approaches are trained on gene expression data, there will be overlap in genes used to train both classifiers and, consequently, overlap in cell type prediction. Yet, we found different genes to be differentially expressed between cancer cell clusters and MSI-H and MSS cells. This is likely because there is no overlap in training data between the two tools. One other factor to consider is that we found a loss of microsatellite instability signal in MSI-H individuals after subsetting down to the cancer cells. Despite being necessary at the single-cell level to only label cells as MSI-H if they were also determined to be cancerous by scATOMIC, there were likely instances where MSIsensor-RNA correctly identified MSI-H cells, and scATOMIC did not. Going forward, it would be beneficial for a benchmarking study to be done to determine if MSIsensor-RNA could also better identify cancer cells in MSI-H individuals. Another factor to consider is that there can be an overlap between the genes used in the clustering of cells and the genes used to generate an MSI score. Whether one or more of the 100 genes used in the MSIsensor-RNA baseline are included in the 2,000 most variable genes used in clustering steps of pipeline will change from individual to individual. While not included in this study, we have checked clustering of cancer cells with and without the 100 genes used by MSIsensor-RNA and found it did not appear to affect the clustering results.

While other MSI detection tools exist, we chose to use MSIsensor-RNA, which infers MSI status based on gene expression, as we were using single-cell RNA sequencing data. MSI is typically detected in NGS data by comparing the distribution of indels in microsatellites between a paired-normal and tumor sample. However, the tool we used, MSIsensor-RNA, does not directly detect MSI with microsatellites but instead uses machine learning models trained on gene expression patterns from MSI-H and MSS individuals. This technique is better suited to detect dMMR, which is traditionally measured with IHC staining of genes involved in the mismatch repair pathway. Furthermore, we have shown in a previous study that RNA-based detection methods have a lower performance than DNA-based detection methods on bulk sequencing data.^44^ However, the authors of MSIsensor-RNA report high performance on single-cell RNA sequencing data,^45^ and we found that it could broadly distinguish between the individuals deemed MSI-H and MSS with PCR/IHC tests in our dataset (Figures 1A and 1B; Figures S1A and S1B). Based on these factors, it would be worthwhile to reproduce our results with data generated from other single-cell sequencing technologies, such as whole-genome amplification and sequencing, which would permit the use of more well-known and established NGS tools that measure differences in microsatellite repeats, such as MANTIS and MSIsensor.^46^^,^^47^

Altogether, we found that heterogeneity in microsatellite instability is more common than previously reported, and we found it both in MSI-H and MSS individuals. These results could help to explain why there are reports of treatment resistance and low response rates in MSI-H cancers treated with ICI therapy; however, our study only analyzed single-cell RNA sequencing data from 49 individuals who underwent 3′ and 5′ single-cell RNA sequencing. Further studies are warranted to determine the frequency of heterogeneity in this biomarker at the population level and whether the presence of MSI-H and MSS subclones can have clinical impacts, including the capacity for the rapid evolution of resistance to treatments for which MSI-H is used as a biomarker.

### Limitations of the study

The primary limitation of this study is the relatively small number of individuals who had publicly available single-cell sequencing data with paired clinical MSI status. Although our study consisted of 134 single-cell RNA sequencing samples, these were from only 49 distinct individuals. This limited our ability to assess the frequency of heterogeneity in this biomarker in the general population. In order to better gauge the frequency of heterogeneity in MSI status at the population level, the results found in our study would need to be replicated in a large cohort-based study. Additionally, our study did not have sufficient clinical metadata to establish whether ITH in MSI has clinical implications, and we therefore did not aim to address this subject. Although some individuals in our study did receive ICI treatment, there was no information on when the sample was collected or when treatment had been administered. Future work in this area would need to include such metadata to unravel whether individuals respond differently to treatment if they have a tumor with MSI-H and MSS subclones.

## Resource availability

### Lead contact

Requests for further information should be directed to the lead contact, Cathal Seoighe (cathal.seoighe@universityofgalway.ie).

### Materials availability

This study did not generate new unique reagents.

### Data and code availability

• Data: This article analyzes existing, publicly available data, accessible from either the European Genome-phenome Archive, the Sequence Read Archive, or the Gene Expression Omnibus (key resources table).
• Code: All original code and results have been deposited to Zenodo (key resources table). A distributable version of the computational pipeline used in this study, SINGLE-MSI, is also available via Zenodo (key resources table). We have written the entire workflow in Snakemake to ensure reproducibility and scalability.
• Other: Any additional information required to analyze the data reported in this article is available from the lead contact upon request.

## Acknowledgments

We would like to thank the patients and researchers who made this study possible by sharing their data. This includes patients from the CRC-SG1, KUL3, and KUL5 cohorts in Joanito et al., patients involved in the PICC study (NCT03926338) from Li et al., and the 6 individuals from Yunnan Cancer Hospital from Wu et al. We would also like to thank Micheál Ó Dálaigh for useful conversations on navigating single-cell cancer data and Anna Groβbach for advice on figure design. This research was funded by Research Ireland through the Research Ireland Centre for Research Training in Genomics Data Science under Grant number 18/CRT/6214. Lastly, we would like to thank Dr. Eleanor Jayawant for making the original cell figures used in the first panel of our graphical abstract available through the following license: CC-BY 4.0 Unported https://creativecommons.org/licenses/by/4.0/.

## Author contributions

Conceptualization, H.A. and C.S.; methodology, H.A. and C.S.; investigation, H.A.; writing – original draft, H.A.; writing – review and editing, H.A. and C.S.; funding acquisition, C.S.; resources, C.S.; supervision, C.S.

## Declaration of interests

The authors declare no competing interests.

## STAR★Methods

### Key resources table

**Table:** 

REAGENT or RESOURCE	SOURCE	IDENTIFIER
**Deposited data**		

Previously published single-cell RNA sequencing data	Joanito et al.^52^	EGA: EGAD00001008555, EGAD00001008584, EGAD00001008585
Previously published single-cell RNA sequencing data	Li et al.^53^	GEO: GSE205506
Previously published single-cell RNA sequencing data	Wu et al.^54^	SRA: PRJNA932556
SINGLE-MSI Pipeline manuscript results	This paper	Zenodo: https://doi.org/10.5281/zenodo.18249691

**Software and algorithms**		

Cell Ranger version 7.2.0	10X Genomics	https://www.10xgenomics.com/support/software/cell-ranger/downloads/
Conda version 24.1.2	Anaconda	https://anaconda.org/anaconda/conda
InferCNV version 1.20.0	Tickle et al.^55^	https://anaconda.org/channels/bioconda/packages/bioconductor-infercnv/files
MSIsensor-RNA version 0.1.6	Jia et al.^45^	https://anaconda.org/channels/bioconda/packages/msisensor-rna/overview
scATOMIC version 2	Nofech-Mozes et al.^56^	https://github.com/abelson-lab/scATOMIC
SINGLE-MSI Pipeline	This paper	Zenodo: https://doi.org/10.5281/zenodo.18250137
Snakemake version 8.27.1	Mölder et al.^34^	https://snakemake.github.io/
R version 4.3.3	R CoreTeam	https://www.r-project.org/
R package caret version 7.0-1	Kuhn^57^	https://cran.r-project.org/web/packages/caret/index.html
R package ggplot2 version 3.5.1	Wickham^58^	https://cran.r-project.org/web/packages/ggplot2/index.html
R package MLeval version 0.3	CRAN	https://cran.r-project.org/web/packages/MLeval/index.html
R package Seurat version 5.1.0	Hao et al.^59^	https://cran.r-project.org/web/packages/Seurat/index.html
R package stats version 1.3.0	R Studio Team	https://cran.r-project.org/doc/manuals/r-patched/packages/stats/refman/stats.html

### Experimental model and study participant details

We used single-cell RNA sequencing data that was generated as part of three previous studies^52^^,^^53^^,^^54^ (Table 2, key resources table). Raw FASTQ files were downloaded from either the European Genome-Phenome Archive or from the Sequence Read Archive (key resources table). All other data was downloaded in matrix format from the Gene Expression Omnibus (key resources table). The data consists of 134 samples from 49 individuals with metastatic or non-metastatic colorectal cancer. Individuals were grouped into MSI-H and MSS categories based on the original PCR/IHC clinical status reported in previous studies. In total there were 29 deemed MSI-H, 18 MSS, and two did not have a reported MSI status (Table 1). Each sample was created with either Single Cell 3’ v2, 3’ v3, or 5’ Reagent Kit from 10X Genomics and was sequenced either on an Illumina NextSeq 500, NovaSeq 6000, BGISEQ DNBSEQ-T7, or HiSeq X Ten machine. Complete sequencing and library preparation information can be found by referencing the Dataset ID in Table 2.

**Table 2:** Single-cell sequencing datasets

Dataset ID	Cancer type	Individuals	Samples	Sequencing
EGA: EGAD00001008555	Colorectal/metastatic	15	77	Illumina HiSeq 4000
EGA: EGAD00001008584	Colorectal/metastatic	3	6	Illumina HiSeq 4000
EGA: EGAD00001008585	Colorectal/metastatic	6	18	Illumina NextSeq 500/NovaSeq 6000
GEO: GSE205506	Colorectal	19	27	Illumina NovaSeq 6000/DNBSEQ-T
SRA: PRJNA932556	Colorectal	6	6	HiSeq X Ten

A table detailing each dataset used in the study. The Dataset ID column specifies the referential ID for each study cohort, European Genome-phenome Archive (EGA prefix), Genome Expression Omnibus ID (GSE prefix), or SRA project code (PRJNA prefix).

Individuals from datasets EGAD00001008555, EGAD00001008584, EGAD00001008585 had multi-regional samples from the same tumor and multi-site samples from metastatic tissue and lymph nodes. Although variation in the multi-site samples could be considered intra-individual heterogeneity rather than ITH, we kept them in the analysis to retain as many cancer cells and as much heterogeneity as possible. The other two datasets GSE205506 and PRJNA932556 include individuals that had treatment for MSI (anti-PD-1 and celecoxib). We excluded the following samples because we did not identify any cancer cells: XHC080-SI-GA-B11, XHC082-SI-GA-C1, XHC127-SI-GA-F10, EXT129, EXT051, and EXT097.

While demographic metadata (age, gender, etc.) was available for most samples, we did not factor this into our analysis and acknowledge it as a limitation. Our study was computational and did not employ a traditional experimental design that would account for these confounding factors. While there is evidence that age, race, and gender affect prognosis and frequency of MSI-H cancer^48^^,^^49^^,^^50^^,^^51^ our study was explorative in nature and did not aim to describe the relationship between these types of variables and the detection of ITH in MSI. As there were very few publicly available single-cell RNA sequencing datasets with paired clinical MSI status at the time we conducted this study, little would have been gleaned from incorporating demographic metadata. We suggested that studies with larger sample sizes would be needed in order to determine the frequency of ITH in MSI at the population level, and those studies would also be needed to investigate such questions related to demographic information.

All ethical approval information for each dataset can be found in the original publications (Table 2, key resources table).

### Method details

#### Data processing

We aligned FASTQ files to the GRCh38 human reference genome and converted them to a gene count matrix using the 10x Genomics Cell Ranger v7.2.0 software suite. From there all matrix files were processed using the R package Seurat^59^ following the Seurat best practices tutorials (https://satijalab.org/seurat/). Briefly, Seurat objects were created and only genes detected in a minimum of 3 cells were used for downstream analysis. We further filtered out cells with fewer than 100 features, more than 3000 features, and if a cell had more than 35 percent of all genes labeled as mitochondrial. While we followed the Seurat best practices closely, the default settings aim to maximize immune cell type identification and filtered out the majority of cancer cells. The filter settings described here were designed to maximize the number of tumor cells retained while still removing cells that were poor-quality or likely necrotic. These filter settings, specifically mitochondrial gene percentage, are supported by a recent study that showed that filter settings that are too strict remove viable cancer cells from single-cell sequencing data.^60^ After filtering, all gene count matrices then were normalized using the LogNormalize option with the scale setting set to 10,000. The 2,000 most variable genes were found using the “vst” selection method and were used to cluster together groups of cells with the RunPCA function. The first 15 PCA dimensions were used to run the following functions: FindNeighbors, FindClusters (resolution set to 0.5), and RunUMAP. If an individual had multiple samples, they were integrated together by using the IntegrateLayers function, with the method set to CCAIntegration and k.weight set to 50. Each integrated sample then underwent re-clustering with the settings previously mentioned. The integration step after subsetting down to only cancer cells used the same settings except the FindClusters resolution was set to 0.8, and only the first 10 principal components were used.

#### Cell classification and measuring ITH

After each sample is processed, all cells are classified as either cancer or normal and then MSI-H or MSS. These classification steps are built upon two machine learning based programs trained on large pan-cancer datasets. The first, scATOMIC,^56^ was used to distinguish tumor cells from normal ones, and the second, MSIsensor-RNA^45^ determined MSI status. Both tools were run with default settings, but to get an MSI score for each cell, we had to transform the prebuilt MSIsensor-RNA baseline file. This was done by filtering both the count matrix and baseline to only include gene names common to both. The filtered baseline and count matrix files were then used to get MSI scores for all cells within a sample. From there cells were classified as MSI-H if they also were labeled as cancer by scATOMIC and if they had an MSI score of .75 or more (75% probability the cell is MSI-H).

Levels of ITH were assessed with two methods. First, we measured ITH by testing for differences in mean MSI score between cancer cell clusters with a one-way ANOVA test. The ANOVA F-statistic was used to describe levels of heterogeneity in the biomarker, with a large value of the F-statistic indicating greater heterogeneity in MSI. Secondly, we identified subclones within each individual by comparing CNVs between MSI-H, MSS, and normal cells. This was done by passing the relevant cell classification for each unique barcode to InferCNV^55^ (https://doi.org/10.18129/B9.bioc.infercnv). InferCNV was used with default settings except in the case of CRC2821, which had many more cancer cells than the other samples. We increased the k_nn setting from the default of 20 to 50 to take into account the larger dataset. Lastly, we ran differential expression using a Wilcoxon Rank Sum Test (the default for Seurat) between clusters of cancer cells and between MSI-H and MSS cancer cells for each individual.

We verified how well our pipeline captured heterogeneity in MSI status by mixing together randomly sampled tumor and normal cells in varying proportions using a custom R script. We simulated varying levels of heterogeneity by mixing the cells of one sample that had homogenous MSI-H cancer cells (GSM6213995 from individual P33) and one sample with homogenous MSS cancer cells (XHC118-SI-GA-F1 from individual CRC2811). In total, we had eleven different mixes, with the proportion of MSI-H cells ranging from 0 to 1 (in increments of 0.1) and the remainder being MSS (Table S1). The results of these mixing experiments were replicated 100 times, except for the pure MSS and MSI-H cases, for which all cancer cells were included.

Although MSIsensor-RNA has been shown to classify single-cell RNA sequencing samples accurately,^45^ we checked its ability to distinguish between MSI-H and MSS samples in our datasets at the individual level. This was done by scoring individuals with MSIsensor-RNA using all available cells and again with just the cancer cells. We used the AggregateExpression function in Seurat to create the two different scenarios, and measured MSIsensor-RNA performance with ROC-AUC using the MLeval and caret packages in R.^57^^,^^61^

### Quantification and statistical analysis

All statistical analyses were carried out in R (R version 4.3.3; https://www.R-project.org/)^62^ and all plots were created with ggplot2.^58^ Two statistical tests were performed as part of our computational pipeline. The first is a one-way ANOVA test that we used to measure ITH by comparing the difference in means between clusters of cancer cells. This was done with the aov function and was followed with Tukey’s Honestly Significant Difference test using the TukeyHSD function, both of which are from the stats package.^62^

## Supplemental information

Document S1. Figures S1–S3 and Tables S1 and S3–S9

Table S2. Mixing experiment raw results, related to Figure 1
