# Supplementary material for: Intratumoral heterogeneity in microsatellite instability status at single-cell resolution
Source: iScience. 2026 Feb 5;29(3):114860. doi: 10.1016/j.isci.2026.114860 (PMC12936829; doi:10.1016/j.isci.2026.114860)
Supplement: Document S1. Figures S1–S3 and Tables S1 and S3–S9 [file mmc1.pdf]

iScience, Volume *29*

Supplemental information

Intratumoral heterogeneity in microsatellite instability status at single-cell resolution

Harrison Anthony and Cathal Seoighe

A

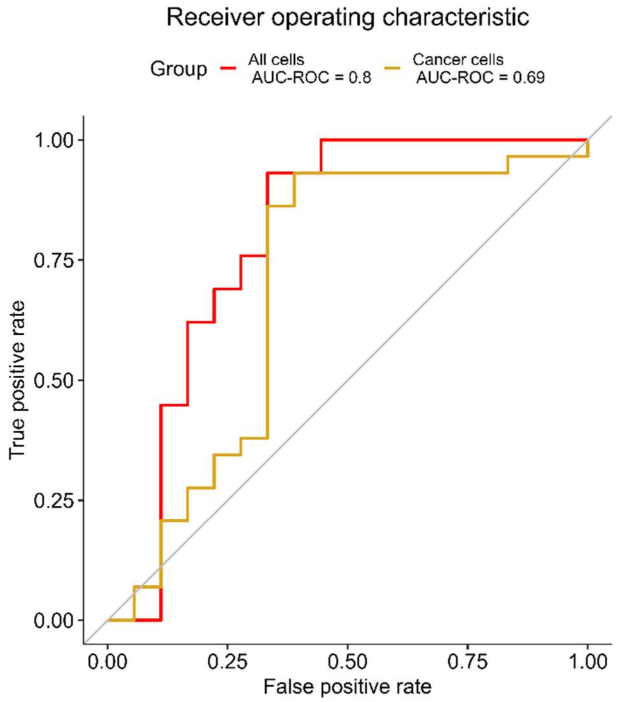

B

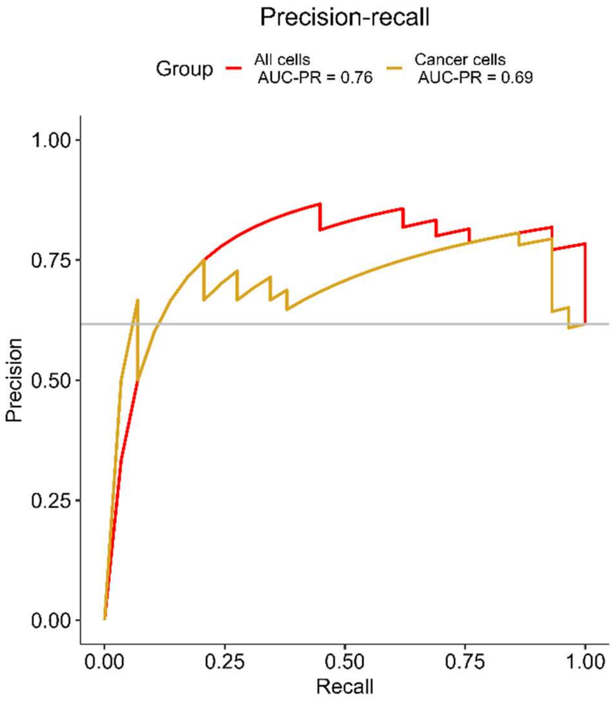

Figure S1. MSIsensor-RNA ROC and precision-recall curves, related to Figure 1. Plots showing performance of MSIsensor-RNA based on (A) ROC and (B) precision-recall curves. Line colors distinguish whether MSIsensor-RNA was run on a dataset consisting of all cells or only cancer cells.

A

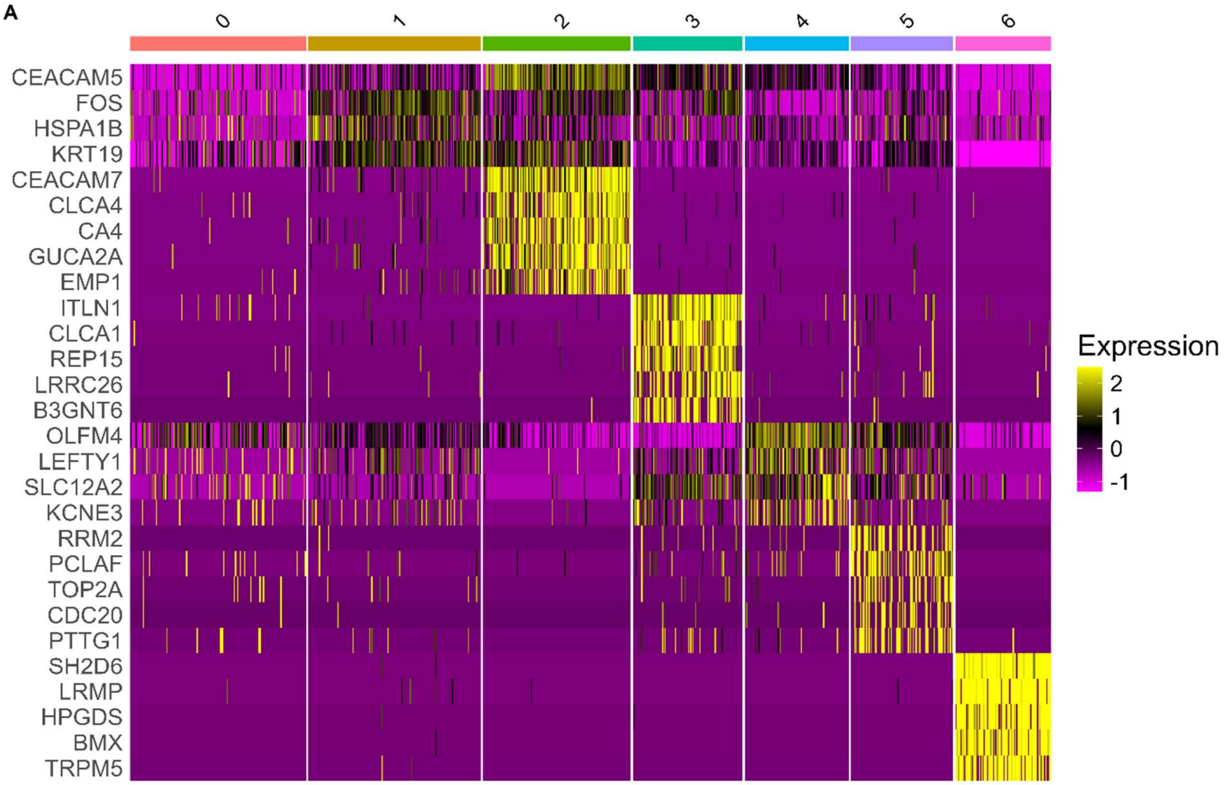

B

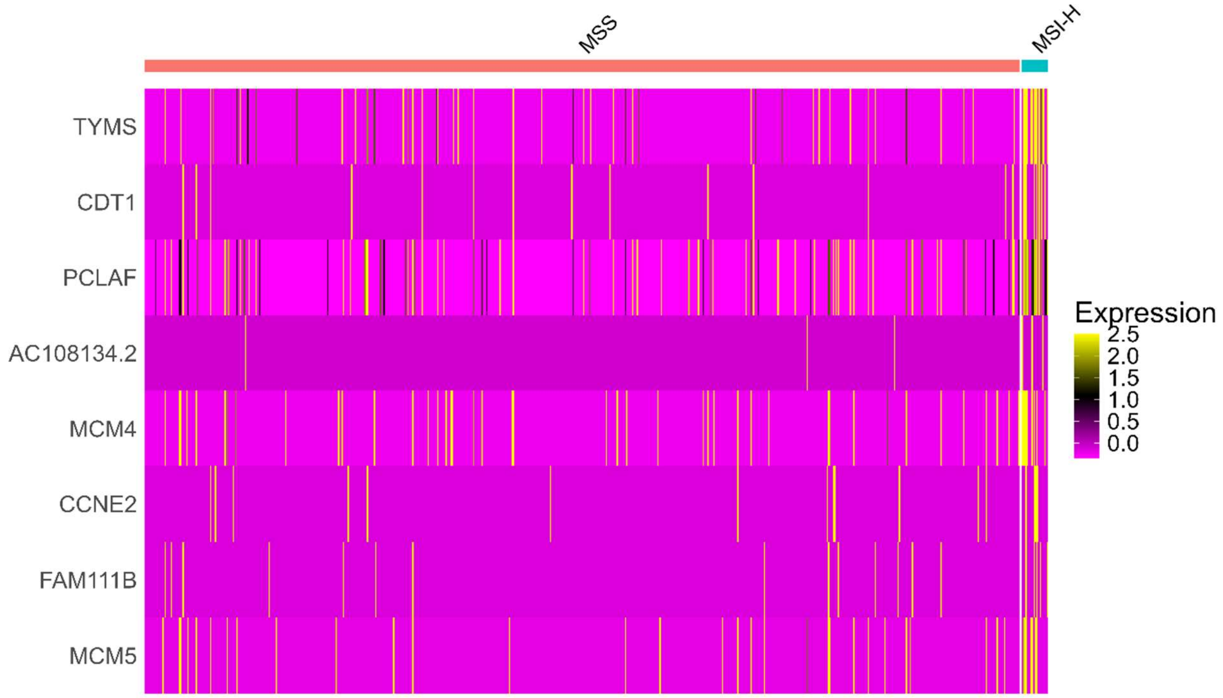

Figure S2. Differential gene expression heatmaps for MSI-H indivdiual, related to Figure 4. Heatmaps of differential gene expression analysis for MSI-H individual, P24. Panel (A) shows differential gene

expression between cancer cell clusters, and panel (B) shows differential gene expression between MSI-H and MSS cells.

A

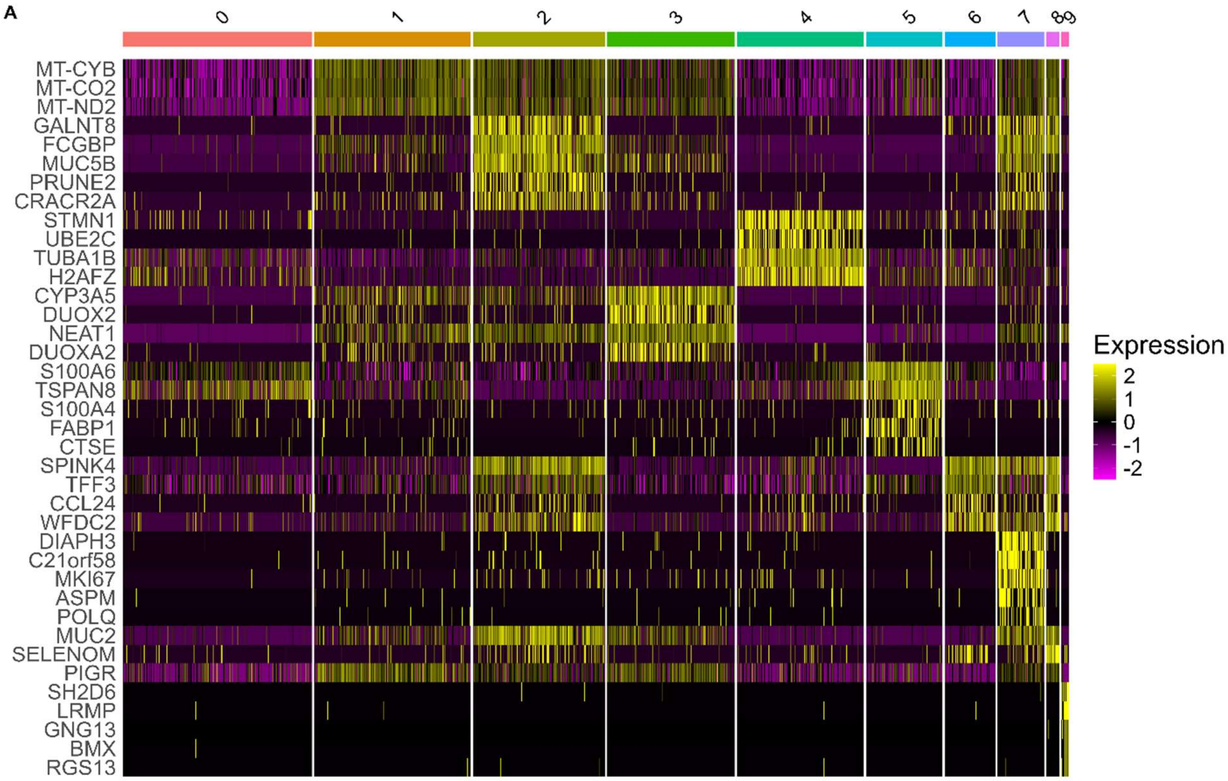

B

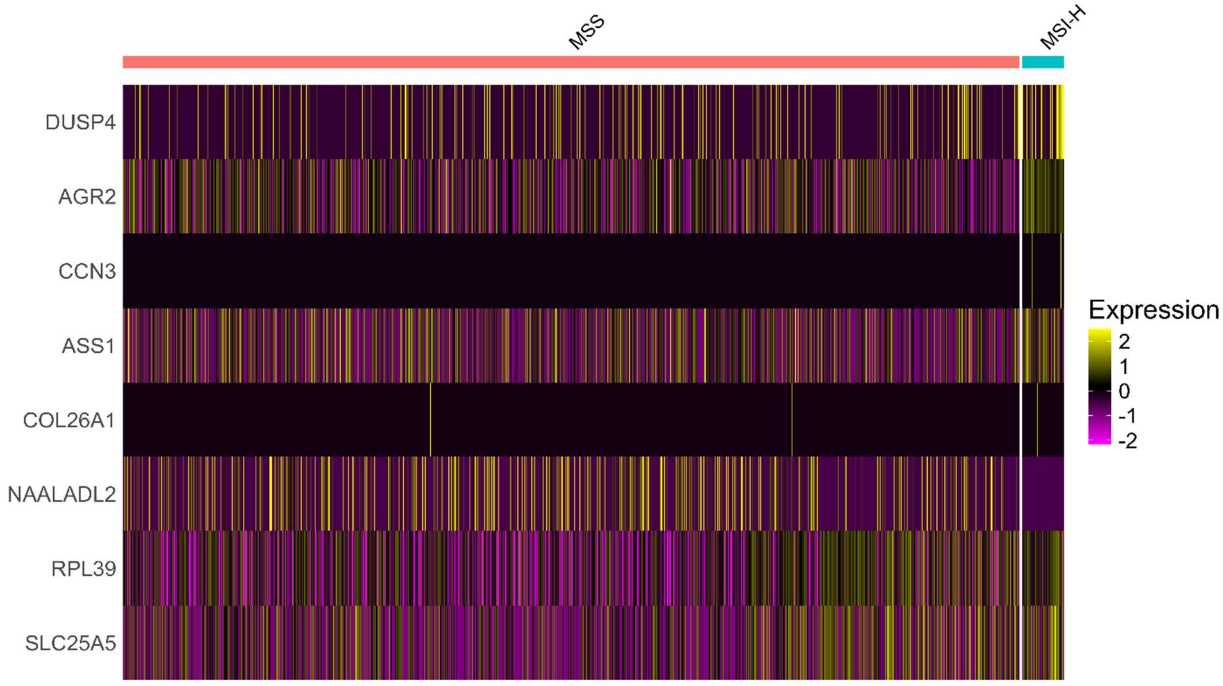

Figure S3. Differential gene expression heatmaps for MSS individual, related to Figure 5. Heatmaps of differential gene expression analysis for MSS individual, CRC2786. Panel (A) shows differential gene

expression between cancer cell clusters, and panel (B) shows differential gene expression between MSI-H and MSS cells.

F SE	F	MSI-H	MSS	MSI-H SE	MSS SE	Mix	Prop MSI-H	Prop MSS
9.56	191.54	0.99	13.39	0.02	0.23	Mix 1	0.10	0.90
15.05	332.59	1.00	11.86	0.00	0.25	Mix 2	0.20	0.80
15.83	382.09	1.00	10.39	0.00	0.24	Mix 3	0.30	0.70
13.49	383.07	1.00	9.30	0.00	0.23	Mix 4	0.40	0.60
13.86	371.92	1.00	8.44	0.00	0.25	Mix 5	0.50	0.50
10.7	318.80	1.00	7.49	0.00	0.19	Mix 6	0.60	0.40
6.86	246.94	1.00	7.22	0.00	0.21	Mix 7	0.70	0.30
6.80	170.56	1.00	7.46	0.00	0.22	Mix 8	0.80	0.20
2.11	81.17	1.00	8.05	0.00	0.19	Mix 9	0.90	0.10
0.00	11.53	0.00	16.00	0.00	0.00	MSS	0.00	1.00
0.00	15.90	1.00	11.00	0.00	0.00	MSI-H	1.00	0.00

Table S1. Mixing experiment summary statistics, related to Figure 1. Summary statistics for all mixing runs reporting the average F-statistic, number of MSI-H subclones, and number of MSS subclones. The MSI-H and MSS mixes are not representative of an average as they are the results for samples used in the mixing experiment. Also reported is twice the standard error (SE) for all the summary statistics.

Individual	Df	Ssq	Msq	F	Adjusted P
CRC2783	3	2.53	0.84	17.15	0.00
CRC2786	9	36.38	4.04	75.67	0.00
CRC2787	1	0.12	0.12	1.62	0.21
CRC2794	5	1.34	0.27	9.51	0.00
CRC2795	5	1.68	0.34	8.17	0.00
CRC2801	6	0.65	0.11	4.66	0.00
CRC2803	7	4.89	0.70	15.91	0.00
CRC2810	2	0.10	0.05	1.80	0.17
CRC2811	7	1.43	0.20	19.63	0.00
CRC2816	6	1.30	0.22	8.68	0.00
CRC2817	6	1.72	0.29	10.62	0.00
CRC2821	11	64.59	5.87	104.26	0.00
CRC2829	8	0.50	0.06	10.04	0.00
CRC2841	7	10.19	1.46	93.35	0.00
CRC2899	8	3.01	0.38	20.28	0.00
P11	2	0.28	0.14	6.94	0.00
P12	2	0.40	0.20	3.02	0.05
P14	0	NA	NA	NA	NA
P15	2	0.19	0.10	2.25	0.11
P17	0	NA	NA	NA	NA
P18	9	12.16	1.35	30.89	0.00
P19	1	0.07	0.07	1.30	0.26
P21	0	NA	NA	NA	NA
P23	9	14.38	1.60	29.07	0.00
P24	6	18.83	3.14	75.20	0.00
P25	6	9.55	1.59	34.94	0.00
P26	5	1.19	0.24	5.09	0.00
P27	4	1.76	0.44	11.26	0.00
P28	4	6.22	1.55	51.15	0.00
P29	4	1.10	0.27	10.99	0.00
P30	5	5.49	1.10	26.69	0.00
P31	9	6.87	0.76	18.71	0.00
P32	6	5.10	0.85	19.41	0.00
P33	5	2.29	0.46	15.71	0.00
SC024	4	4.49	1.12	21.77	0.00
SC027	7	1.44	0.21	20.24	0.00
SC029	3	0.18	0.06	5.78	0.00
SC035	5	12.95	2.59	47.22	0.00
SC040	8	36.40	4.55	116.10	0.00
SC041	4	2.71	0.68	10.01	0.00
SC042	2	2.05	1.03	31.24	0.00
SC043	6	0.52	0.09	6.75	0.00
SC044	7	8.46	1.21	32.87	0.00
SRR23490337	0	NA	NA	NA	NA
SRR23490338	1	0.45	0.45	7.66	0.01
SRR23490339	0	NA	NA	NA	NA
SRR23490340	3	4.36	1.45	27.42	0.00
SRR23490341	2	3.98	1.99	48.02	0.00

SRR23490342	0	NA	NA	NA	NA

Table S3. ANOVA test results for each individual, related to Figure 2. ANOVA test results for each individual. We have abbreviated degrees of freedom to Df, sum of squares to Ssq, mean sum of squares to Msq, F is the ANOVA F-statistic, and Adjusted P is the p-value associated with the test.

Difference	Lower	Upper	Adjusted P	Cluster pair
0.03	-0.04	0.10	0.84	1-0
-0.21	-0.29	-0.14	0.00	2-0
0.21	0.13	0.29	0.00	3-0
0.11	0.03	0.20	0.00	4-0
0.15	0.07	0.23	0.00	5-0
-0.27	-0.36	-0.18	0.00	6-0
-0.25	-0.32	-0.17	0.00	2-1
0.18	0.10	0.26	0.00	3-1
0.08	0.00	0.16	0.05	4-1
0.12	0.04	0.20	0.00	5-1
-0.30	-0.39	-0.22	0.00	6-1
0.43	0.34	0.51	0.00	3-2
0.33	0.24	0.41	0.00	4-2
0.37	0.28	0.45	0.00	5-2
-0.06	-0.14	0.03	0.47	6-2
-0.10	-0.19	-0.01	0.02	4-3
-0.06	-0.15	0.03	0.42	5-3
-0.48	-0.57	-0.39	0.00	6-3
0.04	-0.05	0.13	0.89	5-4
-0.38	-0.48	-0.29	0.00	6-4
-0.42	-0.52	-0.33	0.00	6-5

Table S4. Tukey HSD results for individual P24, related to Figure 4. This table contains the Tukey HSD results for individual P24. The Lower and Upper columns contain values describing the Tukey test boundaries. The Adjusted P column is the p-value associated with the test and “Cluster pair” describes which two clusters are being compared (e.g. 1-0 is between clusters 1 and 0).

Difference	Lower	Upper	Adjusted P	Cluster pair
-0.18	-0.23	-0.13	0.00	1-0
-0.08	-0.14	-0.03	0.00	2-0
-0.27	-0.32	-0.21	0.00	3-0
0.09	0.03	0.14	0.00	4-0
0.03	-0.03	0.10	0.88	5-0
0.10	0.03	0.17	0.00	6-0
-0.15	-0.23	-0.08	0.00	7-0
0.17	0.03	0.30	0.00	8-0
-0.40	-0.57	-0.22	0.00	9-0
0.09	0.04	0.15	0.00	2-1
-0.09	-0.14	-0.03	0.00	3-1
0.27	0.21	0.32	0.00	4-1
0.21	0.14	0.27	0.00	5-1
0.28	0.20	0.35	0.00	6-1
0.03	-0.05	0.10	0.98	7-1
0.35	0.21	0.48	0.00	8-1
-0.22	-0.40	-0.04	0.00	9-1
-0.18	-0.24	-0.13	0.00	3-2
0.17	0.11	0.23	0.00	4-2
0.11	0.05	0.18	0.00	5-2
0.18	0.11	0.26	0.00	6-2
-0.07	-0.15	0.01	0.14	7-2
0.25	0.12	0.39	0.00	8-2
-0.32	-0.49	-0.14	0.00	9-2
0.35	0.29	0.41	0.00	4-3
0.30	0.23	0.36	0.00	5-3
0.37	0.29	0.44	0.00	6-3
0.11	0.04	0.19	0.00	7-3
0.43	0.30	0.57	0.00	8-3
-0.13	-0.31	0.04	0.33	9-3
-0.06	-0.12	0.01	0.22	5-4
0.01	-0.07	0.09	1.00	6-4
-0.24	-0.32	-0.16	0.00	7-4
0.08	-0.05	0.22	0.67	8-4
-0.49	-0.66	-0.31	0.00	9-4
0.07	-0.02	0.15	0.24	6-5
-0.18	-0.27	-0.10	0.00	7-5
0.14	-0.00	0.28	0.06	8-5
-0.43	-0.61	-0.25	0.00	9-5
-0.25	-0.35	-0.16	0.00	7-6
0.07	-0.08	0.21	0.89	8-6
-0.50	-0.68	-0.31	0.00	9-6
0.32	0.17	0.47	0.00	8-7
-0.25	-0.43	-0.06	0.00	9-7
-0.57	-0.78	-0.35	0.00	9-8

Table S5. Tukey HSD results for individual CRC2786, related to Figure 5. This table contains the Tukey HSD results for individual CRC2786. The Lower and Upper columns contain values describing the Tukey test boundaries. The Adjusted P column is the p-value associated with the test and “Cluster pair” describes which two clusters are being compared (e.g. 1-0 is between clusters 1 and 0).

P	Average Log2FC	Percent1	Percent2	Adjusted P	Cluster	Gene
0.00	-2.04	0.05	0.57	0.00	0	BSG
0.00	-2.61	0.09	0.59	0.00	0	AOC1
0.00	-1.06	0.13	0.68	0.00	0	HLA-E
0.00	-1.93	0.20	0.72	0.00	0	CEACAM5
0.00	-1.41	0.07	0.58	0.00	0	ST14
0.00	1.25	0.91	0.46	0.00	1	FOS
0.00	1.39	0.81	0.42	0.00	1	HSPA1B
0.00	1.26	0.83	0.46	0.00	1	STARD10
0.00	1.10	0.74	0.33	0.00	1	CES2
0.00	1.10	0.91	0.67	0.00	1	KRT19
0.00	6.44	0.91	0.05	0.00	2	CEACAM7
0.00	7.09	0.82	0.03	0.00	2	CLCA4
0.00	7.04	0.80	0.03	0.00	2	CA4
0.00	7.37	0.80	0.03	0.00	2	GUCA2A
0.00	5.44	0.71	0.03	0.00	2	EMP1
0.00	5.43	0.92	0.05	0.00	3	ITLN1
0.00	6.94	0.82	0.04	0.00	3	CLCA1
0.00	4.90	0.74	0.02	0.00	3	REP15
0.00	4.12	0.68	0.03	0.00	3	LRRC26
0.00	6.66	0.56	0.01	0.00	3	B3GNT6
0.00	2.49	0.96	0.59	0.00	4	OLFM4
0.00	1.74	0.79	0.25	0.00	4	LEFTY1
0.00	1.82	0.84	0.34	0.00	4	SLC12A2
0.00	2.04	0.52	0.13	0.00	4	KCNE3
0.00	1.21	0.88	0.42	0.00	4	MLEC
0.00	5.62	0.47	0.02	0.00	5	RRM2
0.00	3.18	0.61	0.06	0.00	5	PCLAF
0.00	3.51	0.48	0.03	0.00	5	TOP2A
0.00	5.95	0.38	0.01	0.00	5	CDC20
0.00	4.19	0.50	0.05	0.00	5	PTTG1
0.00	11.53	0.99	0.00	0.00	6	SH2D6
0.00	10.00	0.93	0.01	0.00	6	LRMP
0.00	11.94	0.85	0.00	0.00	6	HPGDS
0.00	12.27	0.84	0.00	0.00	6	BMX
0.00	9.86	0.80	0.00	0.00	6	TRPM5

Table S6. Differential gene expression results between cancer cells for individual P24, related to Figure 4. Results of the differential gene expression analysis between clusters of cancer cells for MSI-H individual, P24. P, is the p-value associated with the test. The column “Average Log2FC” is the Log fold-change of the average expression between the cancer cell cluster (identified in the Cluster column) and all other clusters. The “Percent1” and “Percent2” columns describe the percentage of cells expressing the gene in either the cancer cell cluster or all other clusters, respectively. The ”Adjusted P” column is the p-value after Bonferroni correction.

P	Average Log2FC	Percent1	Percent2	Adjusted P	Cluster	Gene
0.00	1.54	1.00	0.96	0.00	0	EEF1A1
0.00	1.27	0.99	0.93	0.00	0	RPLP0
0.00	1.01	1.00	0.97	0.00	0	RPL41
0.00	1.23	0.99	0.90	0.00	0	RPS3A
0.00	1.40	0.96	0.80	0.00	0	FTL
0.00	1.41	0.99	0.84	0.00	1	MT-CO3
0.00	1.52	0.98	0.77	0.00	1	MT-CYB
0.00	1.33	1.00	0.84	0.00	1	MT-CO1
0.00	1.45	0.99	0.87	0.00	1	MT-CO2
0.00	1.46	0.96	0.64	0.00	1	MT-ND2
0.00	3.04	0.72	0.11	0.00	2	GALNT8
0.00	2.88	0.95	0.34	0.00	2	FCGBP
0.00	2.67	0.92	0.28	0.00	2	MUC5B
0.00	3.70	0.56	0.07	0.00	2	PRUNE2
0.00	2.90	0.71	0.14	0.00	2	CRACR2A
0.00	3.62	0.77	0.16	0.00	3	STMN1
0.00	5.48	0.47	0.03	0.00	3	UBE2C
0.00	2.98	0.94	0.52	0.00	3	TUBA1B
0.00	3.24	0.83	0.28	0.00	3	H2AFZ
0.00	1.48	1.00	0.93	0.00	3	PTMA
0.00	2.71	0.92	0.32	0.00	4	CYP3A5
0.00	3.65	0.61	0.09	0.00	4	DUOX2
0.00	2.04	1.00	0.42	0.00	4	NEAT1
0.00	1.82	1.00	0.61	0.00	4	MALAT1
0.00	3.15	0.56	0.10	0.00	4	DUOXA2
0.00	2.16	1.00	0.96	0.00	5	S100A6
0.00	2.47	0.91	0.46	0.00	5	TSPAN8
0.00	4.45	0.48	0.07	0.00	5	S100A4
0.00	3.98	0.42	0.05	0.00	5	FABP1
0.00	4.47	0.32	0.03	0.00	5	CTSE
0.00	2.00	1.00	0.40	0.00	6	SPINK4
0.00	1.96	1.00	0.85	0.00	6	TFF3
0.00	3.26	0.47	0.09	0.00	6	CCL24
0.00	2.58	0.76	0.32	0.00	6	WFDC2
0.00	1.84	0.94	0.70	0.00	6	SH3BGRL3
0.00	4.95	0.61	0.02	0.00	7	DIAPH3
0.00	5.14	0.62	0.03	0.00	7	C21orf58
0.00	3.63	0.72	0.05	0.00	7	MKI67
0.00	5.05	0.46	0.02	0.00	7	ASPM
0.00	5.30	0.37	0.01	0.00	7	POLQ
0.00	3.27	0.14	0.01	0.00	8	TPSG1
0.00	1.83	0.84	0.41	0.00	8	FCGBP
0.00	2.16	0.88	0.45	0.00	8	MUC2
0.00	2.08	0.48	0.13	0.00	8	SELENOM
0.00	1.71	0.93	0.65	0.00	8	PIGR
0.00	10.90	0.79	0.00	0.00	9	SH2D6
0.00	9.75	0.79	0.00	0.00	9	LRMP
0.00	11.84	0.47	0.00	0.00	9	GNG13
0.00	8.86	0.58	0.00	0.00	9	BMX
0.00	8.94	0.53	0.00	0.00	9	RGS13

Table S7. Differential gene expression results between cancer cells for individual CRC2786, related to Figure 5. Results of the differential gene expression analysis between clusters of cancer cells for MSS individual, CRC786. P, is the p-value associated with the test. The column “Average Log2FC” is the Log fold-change of the average expression between the cancer cell cluster (identified in the Cluster column) and all other clusters. The “Percent1” and “Percent2” columns describe the percentage of cells expressing the gene in either the cancer cell cluster or all other clusters, respectively. The ”Adjusted P” column is the p-value after Bonferroni correction.

P	Average Log2FC	Percent1	Percent2	Adjusted P	Cluster	Gene
0.00	-3.80	0.06	0.64	0.00	MSS	TYMS
0.00	-2.58	0.02	0.36	0.00	MSS	CDT1
0.00	-2.55	0.04	0.46	0.00	MSS	GINS2
0.00	-6.69	0.00	0.09	0.00	MSS	AC124045.1
0.00	-2.12	0.11	0.68	0.00	MSS	PCLAF
0.00	-2.86	0.03	0.32	0.00	MSS	MCM8
0.00	-2.06	0.01	0.23	0.00	MSS	ASF1B
0.00	-5.37	0.00	0.14	0.00	MSS	AC108134.2
0.00	-5.41	0.00	0.09	0.00	MSS	TAF1A-AS1
0.00	-5.64	0.00	0.09	0.00	MSS	UPK3A
0.00	-5.50	0.00	0.09	0.00	MSS	CBLN3
0.00	-3.38	0.06	0.41	0.00	MSS	MCM4
0.00	-4.45	0.02	0.23	0.00	MSS	AC124067.4
0.00	-3.00	0.03	0.27	0.00	MSS	PYROXD2
0.00	-3.00	0.03	0.27	0.00	MSS	RAD51AP1
0.00	-3.30	0.02	0.23	0.00	MSS	CCNE2
0.00	-3.99	0.02	0.23	0.00	MSS	FAM111B
0.00	-3.25	0.04	0.32	0.00	MSS	MCM5
0.00	-2.79	0.07	0.41	0.00	MSS	OXCT1
0.00	-2.37	0.02	0.23	0.00	MSS	WDHD1
0.00	-2.37	0.06	0.36	0.00	MSS	GLIPR2
0.00	-3.66	0.01	0.14	0.00	MSS	HSPA4L
0.00	-3.29	0.01	0.14	0.00	MSS	ARHGAP33
0.00	-6.38	0.00	0.04	0.00	MSS	CAMTA1-DT
0.00	-6.18	0.00	0.04	0.00	MSS	TNFRSF9
0.00	-6.56	0.00	0.04	0.00	MSS	SLFNL1
0.00	-6.00	0.00	0.04	0.00	MSS	BEND5
0.00	-6.00	0.00	0.04	0.00	MSS	KANK4
0.00	-7.04	0.00	0.04	0.00	MSS	AC234582.1
0.00	-6.25	0.00	0.04	0.00	MSS	AIM2
0.00	-6.18	0.00	0.04	0.00	MSS	RCSD1
0.00	-6.18	0.00	0.04	0.00	MSS	XCL1
0.00	-6.20	0.00	0.04	0.00	MSS	AL355482.2
0.00	-6.18	0.00	0.04	0.00	MSS	AL592148.3
0.00	-6.27	0.00	0.04	0.00	MSS	ZNF670-ZNF695
0.00	-6.18	0.00	0.04	0.00	MSS	AC092580.2
0.00	-8.92	0.00	0.04	0.00	MSS	GNLY
0.00	-5.90	0.00	0.04	0.00	MSS	AC009309.1
0.00	-6.00	0.00	0.04	0.00	MSS	ADRA2B
0.00	-6.18	0.00	0.04	0.00	MSS	ITPRIPL1
0.00	-6.06	0.00	0.04	0.00	MSS	AC009948.3
0.00	-8.64	0.00	0.04	0.00	MSS	NRP2
0.00	-6.18	0.00	0.04	0.00	MSS	COLQ
0.00	-6.80	0.00	0.04	0.00	MSS	PARP15
0.00	-6.80	0.00	0.04	0.00	MSS	SLC9A9
0.00	-6.20	0.00	0.04	0.00	MSS	CXCL5
0.00	-6.56	0.00	0.04	0.00	MSS	AC109361.1
0.00	-6.18	0.00	0.04	0.00	MSS	GZMK

0.00	-6.06	0.00	0.04	0.00	MSS	PDE8B
0.00	-8.14	0.00	0.04	0.00	MSS	AFAP1L1

Table S8. Differential gene expression results between MSI-H and MSS cells for individual P24, related to Figure 4. Results of the differential gene expression analysis between MSI-H and MSS cells for MSI-H individual, P24. P, is the p-value associated with the test. The column “Average Log2FC” is the Log fold-change of the average expression between the cancer cell cluster (identified in the Cluster column) and all other clusters. The “Percent1” and “Percent2” columns describe the percentage of cells in which the gene is detected, where Percent1 will describe the cell type identified in the Cluster column. The ”Adjusted P” column is the p-value after Bonferroni correction.

P	Average Log2FC	Percent1	Percent2	Adjusted P	Cluster	Gene
0.00	-3.06	0.05	0.27	0.00	MSS	UBE2L6
0.00	-3.03	0.03	0.19	0.00	MSS	ENO2
0.00	-2.29	0.11	0.39	0.00	MSS	DUSP4
0.00	-0.56	0.81	1.00	0.00	MSS	AGR2
0.00	-3.75	0.00	0.03	0.00	MSS	S100A3
0.00	-2.65	0.04	0.19	0.00	MSS	TYMS
0.00	-8.44	0.00	0.02	0.00	MSS	CFHR3
0.00	-7.33	0.00	0.02	0.00	MSS	CCN3
0.00	-0.79	0.71	0.88	0.00	MSS	SH3BGRL3
0.00	-0.85	0.52	0.77	0.00	MSS	ASS1
0.00	-0.83	0.48	0.73	0.00	MSS	ALDOA
0.00	-0.54	0.98	1.00	0.00	MSS	GAPDH
0.00	-0.93	0.46	0.75	0.00	MSS	RAN
0.00	-2.39	0.06	0.20	0.00	MSS	OXCT1
0.00	1.39	0.68	0.50	0.00	MSS	MALAT1
0.00	-1.20	0.22	0.45	0.00	MSS	STOML2
0.00	-0.54	0.79	0.95	0.00	MSS	EEF2
0.00	-0.91	0.26	0.52	0.00	MSS	PPP1CA
0.00	-1.22	0.04	0.17	0.00	MSS	SNRNP27
0.00	-4.70	0.00	0.03	0.00	MSS	AL390195.1
0.00	-0.85	0.43	0.66	0.00	MSS	SUMO2
0.00	-0.61	0.85	0.97	0.00	MSS	RPL22
0.00	-2.55	0.00	0.03	0.00	MSS	COL26A1
0.00	-0.57	0.82	0.98	0.00	MSS	RPSA
0.00	-0.61	0.94	1.00	0.00	MSS	ACTB
0.00	-0.86	0.24	0.48	0.00	MSS	TMED2
0.00	-1.23	0.23	0.46	0.00	MSS	NPC2
0.00	4.49	0.26	0.03	0.00	MSS	NAALADL2
0.00	-0.81	0.52	0.77	0.00	MSS	SEM1
0.00	-0.77	0.38	0.61	0.00	MSS	CD63
0.00	-4.82	0.00	0.02	0.00	MSS	LINC01645
0.00	-5.01	0.00	0.02	0.00	MSS	AL445222.1
0.00	-6.13	0.00	0.02	0.00	MSS	FBXO15
0.00	-4.93	0.00	0.02	0.00	MSS	AL160408.3
0.00	-0.55	0.96	1.00	0.00	MSS	EEF1A1
0.00	-0.89	0.31	0.54	0.01	MSS	POMP
0.00	-0.75	0.48	0.72	0.01	MSS	COX7A2
0.00	-0.49	0.89	0.99	0.01	MSS	RPL39
0.00	-0.46	0.96	1.00	0.01	MSS	RPL10
0.00	-0.50	0.78	0.94	0.01	MSS	EEF1G
0.00	-0.49	0.90	0.98	0.01	MSS	RPS6
0.00	-0.80	0.56	0.77	0.01	MSS	SLC25A5
0.00	-0.46	0.90	0.99	0.01	MSS	RPL26
0.00	-0.43	0.96	1.00	0.01	MSS	RPS8
0.00	3.06	0.27	0.05	0.00	MSI-H	UBE2L6
0.00	3.03	0.19	0.03	0.00	MSI-H	ENO2
0.00	2.29	0.39	0.11	0.00	MSI-H	DUSP4
0.00	0.56	1.00	0.81	0.00	MSI-H	AGR2
0.00	3.75	0.03	0.00	0.00	MSI-H	S100A3

0.00	2.65	.019	0.04	0.00	MSI-H	TYMS

Table S9. Differential gene expression results between MSI-H and MSS cells for individual CRC2786, related to Figure 5. Results of the differential gene expression analysis between MSI-H and MSS cells for MSS individual, CRC2786. P, is the p-value associated with the test. The column “Average Log2FC” is the Log fold-change of the average expression between the cancer cell cluster (identified in the Cluster column) and all other clusters. The “Percent1” and “Percent2” columns describe the percentage of cells in which the gene is detected, where Percent1 will describe the cell type identified in the Cluster column. The ”Adjusted P” column is the p-value after Bonferroni correction.
